# Antibiotic resistance and *mec*A characterization of coagulase-negative staphylococci isolated from three hotels in London, UK

**DOI:** 10.3389/fmicb.2015.00947

**Published:** 2015-09-09

**Authors:** Zhen Xu, Hermine V. Mkrtchyan, Ronald R. Cutler

**Affiliations:** School of Biological and Chemical Sciences, Queen Mary University of LondonLondon, UK

**Keywords:** antibiotic resistance, coagulase-negative staphylococci, *mecA* gene, SCC*mec* typing, MLST

## Abstract

Antibiotic resistance in bacteria isolated from non-healthcare environments, is a potential problem to public health. In our survey a total of 71 coagulase negative staphylococci (CNS) belonging to 11 different species were isolated from three large hotels in London, UK. The most prevalent species was *Staphylococcus haemolyticus*, with *S. hominis, S. warneri, S. cohnii*, and *Staphylococcus epidermidis* commonly detected. Antimicrobial susceptibilities and carriage of the *mecA* gene were determined for all of these isolates. Most (85.9%) staphylococci were resistant to multiple antibiotics with all displaying increased susceptibility toward penicillin, fusidic acid, erythromycin, and cefepime. Twenty-one (29.5%) of the isolates were *mecA* positive, however MIC values to oxacillin, normally associated with the carriage of *mecA*, varied widely in this group (from 0.06 to 256 mg/L). Fifteen of the twenty-one *mecA* positive isolates carried SCC*mec* of these seven were type V, one type I, one type II, and one type IV. Additionally, five of these 15 isolates carried a previously unreported type, 1A, which involves an association between class A *mec* complex and *ccr* type 1. The remaining six of the 21 isolates were non-typeable and carried a combination of class A *mec* complex and *ccrC*. In addition to this, we also report on new MLST types which were assigned for five *S. epidermidis* isolates. Four out of these five isolates had MICs between 0.06 and 256 mg/L to oxacillin and would be regarded as clinically susceptible but one isolate had a high oxacillin MIC of 256 mg/L. We demonstrated widespread multiple drug resistance among different staphylococcal species isolated from non-healthcare environments highlighting the potential for these species to act as a reservoir for methicillin and other forms of drug resistance.

## Introduction

Coagulase-negative staphylococci (CNS) are opportunistic pathogens that have emerged as a major cause of nosocomial infections often associated with healthcare settings (Bouchami et al., 2011a; Zong et al., 2011). *S. epidermidis, S. haemolyticus*, and *S. hominis* are amongst the CNS which can be responsible for a wide range of illnesses from minor skin infections to life threatening diseases (Huebner and Goldmann, 1999; Basaglia et al., 2003). In addition to this, the treatment of CNS infections has become more difficult, as many isolates in hospitals carry multiple drug resistance (Bouchami et al., 2011b) due to an increase of ineffectiveness of a wide range of antibiotics (John and Harvin, 2007). Methicillin resistance is commonly associated with the carriage of the *mecA* gene that encodes for penicillin binding protein PBP2a and has a low affinity for β-lactam antibiotics (Tulinski et al., 2012). The *mecA* gene is located on a mobile genetic element called the staphylococcal cassette chromosome *mec* (SCC*mec*) (Milheiriço et al., 2007). There are 11 SCC*mec* types currently reported and typing is based on different combinations of *mec* types (A, B, C1, C2, and D) and *ccr* types (*ccrAB1, ccrAB2, ccrAB3, ccrAB4, and ccrC*) (IWG-SCC, 2009; Shore et al., 2011).

The spread of methicillin resistant *S. aureus* (MRSA) outside of hospital settings, has been reported by several authors and their studies demonstrated that non-healthcare settings, including fire stations, student housing sites/common areas, public buses can be reservoir for MRSA (Roberts et al., 2011a,b; Simões et al., 2011). We have previously reported on the isolation of methicillin resistant coagulase negative staphylococci (MRCNS) from non-hospital restrooms (Mkrtchyan et al., 2013). In this current study we report on the wide variety of antibiotic resistance patterns and the molecular characterization of *mecA* positive determinants that we have isolated from non-healthcare environments.

## Materials and methods

### Screening of staphylococcal isolates

With the permission of the hotel management/owners, we sampled three hotels in London, UK. The results from each hotel were reported to each manager/owner for their information. The sampling was of inanimate objects only. Eighty-eight randomly selected sites in 32 different hotel rooms were sampled using COPAN dry swabs (Copan Diagnostics Inc., USA).

In addition to these samples, 12 air samples were also collected from 12 randomly selected rooms using a high volume air sampler (Cherwell SAS Super 100, Cherwell Laboratories, UK). The airflow of sample collection was between 200 and 1000 l per min.

All specimens were inoculated onto Nutrient Agar (Nutrient Agar, Oxoid, Basingstoke, UK) and Mannitol Salt Agar plates (Oxoid Basingstoke, UK). These cultures were incubated aerobically at 37°C for 24–72 h.

### Identification of environment staphylococci

All isolates were initially screened using conventional methods including microscopy (Gram film), catalase and coagulase testing. Prolex™ staph latex kits (ProLab Diagnostics, Neston, UK) were used to identify CNS and *S. aureus*. All potential staphylococcal isolates were then fully identified using a Matrix-assisted laser desorption ionization time flight mass-spectroscopy (MALDI-TOF-MS, Microflex LT, Bruker Daltonics, Coventry, UK) in a positive linear mode (2000–20,000 m/z range). Samples were prepared as described previously (Mkrtchyan et al., 2013). In brief, 3–5 colonies of fresh cultures were added into 300 μl distilled water and mixed with 900 μl absolute ethanol. The suspension was centrifuged for 2 min at 13,000 × g and the pellets were re-suspended in 25 μl of 70% formic acid and then mixed with 25 μl pure acetonitrile. The mixture was centrifuge for 2 min at 13,000 × g. One microliter aliquots of the obtained supernatant were spotted on the MALDI target plate and overlaid with 1 μl of α-Cyano-4-hydroxycinnamic acid (HCCA) matrix (Bruker Daltonics, Coventry, UK). The resulting spectra for each isolate was analyzed by MALDI-Biotyper 3.0 software (Bruker Daltonics, Coventry, UK). *Escherichia coli* DH5α (Bruker Daltonics, Coventry, UK) was used as a standard for calibration and quality control.

### Antimicrobial susceptibility testing for all staphylococci

The susceptibility of 12 antibiotics was tested using standard disk diffusion method as previously described (Andrews and Howe, 2011). This included amoxicillin (10 μg); cefepime (30 μg); chloramphenicol (30 μg); erythromycin (5 μg); fusidic acid (10 μg); gentamicin (10 μg); mupirocin (20 μg); oxacillin (1 μg); penicillin (1 unit); streptomycin (10 μg); tetracycline (10 μg); vancomycin (5 μg). The categories susceptible, intermediate resistant or resistant were assigned on the basis of the Guidelines for Susceptibility Testing (Andrews and Howe, 2011). The Minimum Inhibitory Concentrations (MIC) for oxacillin were additionally evaluated using “M.I.C. evaluators” (Oxoid Ltd., Basingstoke, UK).

### Detection of *mecA* gene and staphylococci cassette chromosome *mec* (SCC*mec*) typing

The detection of *mecA* gene was carried out using primers and methods as described previously (Hanssen et al., 2004). SCC*mec* typing was performed to *mecA* positive isolates using PCR as described previously, and the isolates were investigated using primers for *mec* and *ccr* complexes (Kondo et al., 2007).

### MLST typing of *Staphylococcus epidermidis*

*S. epidermidis* isolates were further analyzed by Multi Locus Sequence Typing (MLST) for seven housekeeping genes, as has been described previously (Thomas et al., 2007). Sequence types were determined by comparing the alleles to those in the *S. epidermidis* database (www.mlst.net).

Amplicons were sequenced by Eurofins MWG GmBH (Ebersberg, Germany) using ABI 3730XL DNA analyser. Sequence similarity searches were carried out using BLAST tool (http://www.ncbi.nlm.nih.gov/BLAST).

### Southern blotting of *mecA* positive isolates with low oxacillin MIC

*S. hominis* which had the lowest MIC to oxacillin was selected for southern blotting. *S. aureus* NCTC6571 was used as a negative control. *ClaI*-digested genomic DNA were transferred to a nitrocellulose membrane (Roche Diagnostics Ltd., West Sussex, UK) as recommended by the manufacturers (GE health care UK Ltd., Buckinghamshire, UK). Hybridization was carried out with a DNA probe for *mecA* at 68°C, and using the DIG luminescence detection kit according to the manufacturer's instructions (Roche Diagnostics Limited, Charles Avenue, Burgess Hill, West Sussex, UK).

## Results

### Sample collection

A total of 71 staphylococcal isolates were recovered in this study, this included 62 from hotel surfaces and nine collected from hotel air samples.

### Identification of environmental staphylococci

We identified 71 staphylococcal isolates as belonging to 11 different staphylococcal species. These included *S. haemolyticus* (*n* = 34), *S. hominis* (*n* = 12), *S. warneri* (*n* = 5), *S. cohnii* (*n* = 5), *S. epidermidis* (*n* = 5), *S. lugdunensis* (*n* = 3), *S. pettenkoferi* (*n* = 2), *S. capitis* (*n* = 2), *S. sciuri* (*n* = 1), *S. pasteuri* (*n* = 1), and *S. equorum* (*n* = 1).

### Antibiotic susceptibilities for all staphylococci

All isolates were tested for their susceptibility to 12 commonly used antibiotics. We found that all isolates showed resistance to at least one antibiotic. In addition to this, 61 (86%) out of 71 staphylococcal isolates were resistant to two or more of the 12 antibiotics evaluated (Tables 1, 2). Fifty-two (73%) staphylococcal isolates were resistant to penicillin and 50 (70%) were resistance to fusidic acid, 35 (49%) to erythromycin, 24 (34%) to amoxycillin, 23 (32%) to tetracycline, 20 (28%) to cefepime, 20 (28%) to vancomycin, 18 (25%) to mupirocin, 14 (20%) to oxacillin, 13 (18%) to chloramphenicol, 8 (11%) to gentamicin, and 7 (10%) to streptomycin. Among all staphylococci species *S. haemolyticus* was predominantly resistant to erythromycin (59%), followed by *S. epidermidis* to amoxicillin 60% and tetracycline 60% respectively and *S. cohnii* to erythromycin (80%), whereas *S. haemolyticus* (ID 54) and *S. pasteuri* (ID 68) were susceptible to almost all antibiotics tested except for streptomycin (Tables 1, 2).

**Table 1:** **Molecular characterisation of antibiotic resistant and ***mecA*** gene positive coagulase negative staphylococci isolated from hotel and air samples**.

**ID**	**Species**	**Source**	**AM**	**CEF**	**CHL**	**ERY**	**FC**	**GEN**	**MUP**	**PG**	**STR**	**TET**	**VAN**	***mecA***	***mec***	***ccr***	**SCC*mec***	**MIC/Ox (mg/L)**
1	*S. cohnii*	HAS	S	R	S	S	R	S	R	R	I	S	S	+	Class C	C	V	2
2	*S. cohnii*	HAS	S	R	R	R	R	S	S	R	R	R	R	+	Class A	C	NT	1
3	*S. epidermidis*	DSH	R	R	S	R	R	R	R	R	I	R	S	+	Class B	2	IV	256
4	*S. epidermidis*	DSH	R	S	S	I	S	S	S	R	S	R	S	+	Class A	1	1A	0.12
5	*S. haemolyticus*	HAS	S	R	S	R	R	S	S	R	R	R	S	+	Class A	C	NT	256
6	*S. haemolyticus*	DSH	S	R	R	R	R	S	R	R	I	S	R	+	Class C	C	V	256
7	*S. haemolyticus*	DSH	R	R	R	S	R	R	S	R	R	R	R	+	Class A	2	II	256
8	*S. haemolyticus*	DSH	R	R	S	R	R	R	S	R	R	S	S	+	Class C	C	V	8
9	*S. haemolyticus*	DSH	R	R	S	R	R	S	S	R	I	S	S	+	Class C	C	V	8
10	*S. haemolyticus*	DSH	S	R	R	S	R	S	R	R	I	R	R	+	Class A	1	1A	1
11	*S. haemolyticus*	HAS	S	S	R	R	R	S	R	R	R	R	R	+	Class C	C	V	0.5
12	*S. haemolyticus*	DSH	R	S	S	S	R	S	S	R	I	S	R	+	Class C	C	V	0.25
13	*S. haemolyticus*	DSH	R	S	S	S	S	S	S	R	I	R	S	+	Class B	1	I	0.12
14	*S. hominis*	DSH	S	S	R	S	R	S	S	R	S	S	R	+	Class A	1	1A	8
15	*S. hominis*	DSH	R	S	S	R	R	S	R	R	S	R	R	+	Class A	1	1A	0.12
16	*S. hominis*	DSH	R	R	S	R	R	S	R	R	I	S	S	+	Class A	1	1A	0.06
17	*S. lugdunensis*	DSH	S	S	S	R	S	S	S	R	I	S	S	+	Class A	C	NT	0.5
18	*S. pettenkoferi*	DSH	R	R	R	R	R	R	S	R	R	R	R	+	Class A	C	NT	8
19	*S. sciuri*	DSH	S	R	R	S	R	S	S	R	I	S	R	+	Class A	C	NT	16
20	*S. warneri*	DSH	S	S	S	R	S	S	S	R	I	S	S	+	Class A	C	NT	0.25
21	*S. warneri*	DSH	S	S	S	S	R	S	S	R	I	S	R	+	Class C	C	V	0.25

*DSH, different sites from hotels; HAS, hotel air samples; AM, amoxicillin (10 μg); CEP, cefepime (30 μg); CHL, chloramphenicol (30 μg); ERY, erythromycin (5 μg); FC, fusidic acid (10 μg); GEN, gentamicin (10 μg); MUP, mupirocin (20 μg); PG, penicillin (1 unit); STR, streptomycin (10 μg); TET, tetracycline (10 μg); VAN, vancomycin (5 μg); R, resistance; S, sensitive; I, intermediate; MIC/Ox, minimum inhibitory concentration Oxacillin; NT, not typeable*.

**Table 2:** **Molecular characterisation of antibiotic resistant but ***mecA*** gene negative, coagulase negative staphylococci isolated from hotel and air samples**.

**ID**	**Species**	**Source**	**AMO**	**CEF**	**CHL**	**ERY**	**FC**	**GEN**	**MUP**	**PG**	**STR**	**TET**	**VAN**	**OX**
22	*S. capitis*	DSH	S	S	S	S	S	S	R	R	S	S	S	S
23	*S. capitis*	DSH	S	S	S	S	R	S	S	R	I	S	S	S
24	*S. cohnii*	DSH	R	S	S	R	R	S	S	R	S	S	R	S
25	*S. cohnii*	DSH	R	S	S	R	S	S	R	R	S	S	S	S
26	*S. cohnii*	DSH	S	S	R	R	R	S	S	R	S	S	S	S
27	*S. epidermidis*	DSH	S	S	S	S	R	S	S	R	I	S	R	S
28	*S. epidermidis*	DSH	R	S	R	R	R	S	S	R	I	R	S	S
29	*S. epidermidis*	DSH	S	R	R	S	R	S	R	R	I	S	R	S
30	*S. equorum*	DSH	S	S	S	S	S	S	S	R	I	S	S	S
31	*S. haemolyticus*	DSH	R	S	S	R	S	R	S	S	I	R	S	S
32	*S. haemolyticus*	DSH	R	R	S	R	R	S	S	R	S	S	S	S
33	*S. haemolyticus*	DSH	S	S	S	R	R	R	R	R	I	S	R	S
34	*S. haemolyticus*	DSH	S	S	S	R	S	S	S	S	I	S	S	S
35	*S. haemolyticus*	DSH	S	S	S	R	R	S	R	R	S	S	S	S
36	*S. haemolyticus*	DSH	S	S	S	R	R	S	R	R	I	S	S	S
37	*S. haemolyticus*	DSH	R	S	S	R	R	S	S	R	I	S	S	S
38	*S. haemolyticus*	DSH	S	R	S	R	R	S	S	S	S	S	R	S
39	*S. haemolyticus*	DSH	S	S	S	R	R	S	S	S	I	S	S	S
40	*S. haemolyticus*	DSH	S	R	S	R	S	S	S	S	I	R	S	S
41	*S. haemolyticus*	DSH	S	S	S	S	R	S	S	R	I	S	R	S
42	*S. haemolyticus*	DSH	S	S	S	S	R	S	S	R	I	S	S	S
43	*S. haemolyticus*	DSH	S	R	R	R	S	S	S	S	I	R	S	S
44	*S. haemolyticus*	DSH	S	S	S	R	S	S	S	S	I	R	R	S
45	*S. haemolyticus*	DSH	R	R	S	S	R	S	S	R	I	R	S	S
46	*S. haemolyticus*	DSH	R	S	S	R	S	S	S	R	I	S	S	S
47	*S. haemolyticus*	DSH	R	S	S	R	R	S	S	R	I	S	R	S
48	*S. haemolyticus*	DSH	S	S	S	S	S	S	S	S	I	R	S	S
49	*S. haemolyticus*	DSH	S	S	S	R	S	S	S	S	I	R	S	S
50	*S. haemolyticus*	DSH	S	S	S	S	R	S	S	R	I	R	S	S
51	*S. haemolyticus*	DSH	S	S	S	S	R	S	R	S	S	S	S	S
52	*S. haemolyticus*	HAS	S	S	S	S	R	R	S	S	I	R	S	S
53	*S. haemolyticus*	HAS	S	R	S	S	R	S	S	S	I	S	S	S
54	*S. haemolyticus*	HAS	S	S	S	S	S	S	S	S	I	S	S	S
55	*S. haemolyticus*	HAS	R	R	R	S	R	S	R	R	R	R	S	S
56	*S. hominis*	DSH	S	S	S	R	R	S	S	S	S	S	S	S
57	*S. hominis*	DSH	S	S	S	R	R	S	R	R	S	S	S	S
58	*S. hominis*	DSH	R	S	S	S	R	S	S	R	I	S	S	S
59	*S. hominis*	DSH	R	S	S	S	S	S	S	R	S	S	S	S
60	*S. hominis*	DSH	S	S	S	S	S	S	S	R	S	S	S	S
61	*S. hominis*	DSH	S	S	S	R	S	S	S	S	S	S	S	S
62	*S. hominis*	DSH	S	S	S	S	R	S	S	R	I	R	S	S
63	*S. hominis*	DSH	S	S	S	I	R	S	S	S	S	S	S	S
64	*S. hominis*	DSH	S	S	S	S	R	S	S	S	S	S	S	S
65	*S. lugdunensis*	DSH	S	S	S	S	R	S	R	R	I	S	S	S
66	*S. lugdunensis*	HAS	S	S	S	S	R	S	S	R	I	S	S	S
67	*S. pettenkoferi*	DSH	S	S	S	S	S	S	S	R	I	S	S	S
68	*S. pasteuri*	DSH	S	S	S	S	S	S	S	S	I	S	S	S
69	*S. warneri*	DSH	R	S	S	S	R	R	R	S	I	S	S	S
70	*S. warneri*	DSH	R	S	S	R	R	S	S	R	I	R	S	S
71	*S. warneri*	DSH	S	S	S	S	R	S	S	R	I	S	R	S

*DSH, different sites from hotels; HAS, hotel air samples; AMO, amoxicillin (10 μg); CEP, cefepime (30 μg); CHL, chloramphenicol (30 μg); ERY, erythromycin (5 μg); FC, fusidic acid (10 μg); GEN, gentamicin (10 μg); MUP, mupirocin (20 μg); OX, Oxacillin (1 μg); PEN, penicillin (1 unit); STR, streptomycin (10 μg); TET, tetracycline (10 μg); VAN, vancomycin (5 μg); R, resistant; S, sensitive; I, intermediate*.

### Determination of MICs to oxacillin and SCC*mec* typing

Twenty-one of the *mecA* positive isolates were multiple drug resistant however we found that susceptibility to oxacillin was highly variable with MICs ranging from 0.06 to 256 mg/L. Carriage of *mecA* did not always result in isolates demonstrating significant levels of resistance to oxacillin. Seven of the 21 isolates which were *mecA* positive were found to have MICs below 0.5 mg/L to oxacillin (Table 1).

SCC*mec* types were assigned in 15 out of the 21 *mecA* positive isolates. Seven isolates harbored SCC*mec* type V (*n* = 7), and one isolate each harbored SCC*mec* type I (*n* = 1), type II (*n* = 1), and type IV (*n* = 1). Five isolates harbored a new SCC*mec* type 1A, which carried combination of class A *mec* complex and *ccr* type 1. The six isolates that were non-typeable, carried a combination of class A *mec* complex and *ccrC* (Table 1).

### MLST typing of *Staphylococcus epidermidis*

MLST typing revealed that all five *S. epidermidis* isolates contained novel MLST types. These types were assigned as ST515, ST516, ST517, ST518, and ST519.

### Southern blotting of *mecA* positive isolates with low oxacillin MIC

There were also seven isolates that were *mecA* positive but had low MICs to oxacillin. In order to fully confirm that these isolates were indeed *mecA* positive, we selected the isolate from this group with the lowest oxacillin MIC (*S. hominis*, isolate 16, oxacillin MIC 0.06 mg/L, Table 1) and confirmed that it was indeed *mecA* positive using Southern blotting.

## Discussion

The potential threat of antibiotic resistance in environmental/non-healthcare associated bacteria is a concern for public health. Most of the studies on methicillin resistance in staphylococci focus on MRSA and CNS strains isolated from hospital patients (Zong et al., 2011; Brennan et al., 2012; Kinnevey et al., 2013). We have previously reported on the high levels of antibiotic resistance that can be found in bacteria isolated from non-healthcare restrooms (Mkrtchyan et al., 2013). The aim of this current study was to evaluate the levels antibiotic resistance, the carriage of *mecA*, and the diversity of SCC*mec* elements in other staphylococci isolated from high-throughput, non-healthcare environments, in this case hotels.

### Bacterial isolates

A total of 71 staphylococcal isolates belonging to 11 different species recovered from hotel rooms. Within this group, we found that 61 out of 71 isolates (85.9%) were resistant to two or more antibiotics, including one isolate was resistant to 10 antibiotics; 1–9 antibiotics, four isolates were resistant to eight antibiotics; 4–7 antibiotics; 5–6 antibiotics; 7–5 antibiotics; 13–4 antibiotics; 14–3 antibiotics and 12–2 antibiotics (Tables 1, 2). In addition to this the MICs to oxacillin varied widely from 0.06 to 256 mg/L, and although *mecA* genes were detected in 21 isolates, seven of these demonstrated only low levels of methicillin resistance. This is the NCCLS standard level for determining resistance in CNS which are *mecA* positive (Hussain et al., 2000).

### SCC*mec* typing

SCC*mec* types were assigned for the 21 isolates mentioned above. Type V was the most common (7 isolates). This is consistent with some previously published works carried out with clinical isolates (Zong et al., 2011). We did however also identify types I, II, and IV in our isolates, whereas types III, VI, VII, VIII, IX, X, and XI were not detected. It has been reported by others that the distribution of SCC*mec* types in MRCNS varies and may depend on the human host and geographical locations of the isolates were obtained from (Oliveira et al., 2006; IWG-SCC, 2009; Zhang et al., 2009; Zong et al., 2011). In addition to this, in previous papers, SCC*mec* types I, II, III, and V were found to be the most common in environmental isolates, such isolates were taken from areas such as public beaches (Soge et al., 2009).

The structural diversity of SCC*mec* has been reported in hospital environments (Ruppé et al., 2009; Barbier et al., 2011; Zong et al., 2011) with high throughputs of patients. It also possible that the SCC*mec* variations we observed were similarly related to the high throughput of people in the hotels tested (Barbier et al., 2011). In addition to this, we found associations between SCC*mec* carriage and certain species, for example SCC*mec* type V was preferentially associated with *S. haemolyticus, S. hominis, S. warneri, S. epidermidis*, and *S. sciuri*. Previously, with clinical isolates, type V SCC*mec* was reported to be associated mainly with *S. haemolyticus* (Zong et al., 2011).

Apart from the variations in the classified SCC*mec* types isolated, we also reported on 11 unclassified SCC*mec* types. Six of these had a combination of class A *mec* complex and *ccrC* and five had a combination of class A *mec* complex and *ccr* type 1. The latter has been reported by other workers to be a new type 1A (Bouchami et al., 2011b).

### MLST types

Finally to emphasis the wide range of genetic variability that exists among these isolates, we also report new MLST types were assigned for five *S. epidermidis* isolates. Interestingly, four of these were determined to be clinically susceptible to oxacillin, whereas one was highly resistant to oxacillin (MIC of 256 mg/L) (Hussain et al., 2000). To date, studies on *S. epidermidis* have been focused on clinical isolates (Li et al., 2009), we believe that this is the first report to include the molecular characterisation of *S. epidermidis* isolated from non-healthcare sources.

In conclusion, public environments are potential reservoirs of multidrug resistant staphylococci. The characterization of an *S. epidermidis* isolate type with an MIC to oxacillin of 256 mg/L is of particular interest and requires further investigation with regard to its classification within the existing SCC*mec* typing system. We also aim in the future to look into the genetics of these isolates for other 11 antibiotics used in this study. Moreover, we have several other studies on-going which are looking at the bacterial populations in non-healthcare environments that will allow us to compare these samples with those from the current as well as previous studies. The dissemination of multidrug resistance in non-healthcare environments is evidence that infection control measures in the hospitals and in public places are ineffective in limiting the spread of such clones and that these environments are a source of antibiotic resistant pathogens.

### Conflict of interest statement

The authors declare that the research was conducted in the absence of any commercial or financial relationships that could be construed as a potential conflict of interest.
